# Supplementary material for: Auger electron emission initiated by the creation of valence-band holes in graphene by positron annihilation
Source: Nat Commun. 2017 Jul 13;8:16116. doi: 10.1038/ncomms16116 (PMC5511367; doi:10.1038/ncomms16116)
Supplement: Supplementary Information [file ncomms16116-s1.pdf]

Reviewers' Comments:

Reviewer #1:
Remarks to the Author:

specific comments

• At pag. 2 of the manuscript, version at my disposal, I do not entirely understand the term "elastic" used in the context of an Auger emission, which is actually an inelastic scattering by definition ("elastic emission of Auger electrons").

• Still at pag. 2, I would reconsider or, at least, soften the claim that “investigations of the spectra of Auger electrons emitted as a result of the filling of valence holes in solids have not been pursued”.
• Direct comparison between theoretical and “as recorded” experimental spectra without background subtraction has been already pursued in the past. Please cite the relevant literature (e.g. see S Taioli et al. Phys. Rep. 493 (5), 237 (2010) and S. Taioli et al. Phys. Rev. B 79 (8), 085432 (2009)).
• At pag. 3 please consider to state more clearly what is meant by: “positron beam with an incident kinetic energy that is below the threshold for all impact induced electron emission”. What exactly do the authors expect for higher energy positrons?
• At pag. 3 again please cancel out the parenthesis “(below 12 eV)”. This number is indeed misleading, in view of the sentence reported immediately after, where the Auger peak occurs at 4 eV.
• At pag. 6 please soften the claim that the agreement between experimental and calculated spectra is “excellent”. In view of the level of theory used, which does not calculate explicitly the Auger matrix element, and superimposes many parametrized function, such as the escape function. Furthermore, the authors cannot claim that phonons do not contribute much to the broadening of the lineshape by simply comparing it with the experimental spectrum. Typically, these vibronic contributions do have an impact on the lineshape progression, possibly here are smeared out by the convolution with the superimposed broadening.
• At pag. 6 and 7, I suppose the values in parenthesis (4 and 0.2) are meant to indicate errors. Please clarify better this point.
• Please avoid the use of “hot holes” to describe sites that are active with respect to Auger transitions.
• At pag. 7, please clarify better the discussion about the assessment of the VVV Auger efficiency with respect to the KVV Auger lines. This is indeed almost an empirical way to deduce this
• In the discussion about the dissociative attachment at pag. 8, please cite the following reference (S Taioli et al. J. Phys. B: Atomic, Molecular and Optical Physics 39 (21), 4379 (2006)), which represents the first successful attempt of estimating the dissociation probability by using a wavepacket approach in a complex triatomic potential.
• The method by Cini and Sawatzsky for calculating Auger lineshapes, using the convolution of the valence density of states, misses completely the information on the transition probability, which would require the calculation of the Auger matrix elements (and thus of the scattering wavefunction). So, this approach for calculating lineshapes, in addition to the authors’ arbitrary choice of neglecting the hole-hole repulsion in the final states, is somehow closer to semi-empirical methods more than a fully ab-initio approach.
• At pag. 11 please correct the sentence stating “core holes in the valence band” as it is a bit misleading.
• In the Supporting Information, Electronic structure section, please add information about i) cell dimensions, possibly by adding a plot of the graphene-copper complex used in this work; ii) the energy cut-off.
• The numeration of some plots in the Supporting Information (S10, S11) looks not correct as well as the reference in the caption of the last picture of the Supporting Information. B.

Final comment The above should clarify that some technical points are missing from this paper, and many others are left unnecessarily vague. I am sorry not be completely positive in this occasion; however after some revisions, I will be happy to warrant the publication of this article in Nature Communications

Reviewer #2:
Remarks to the Author:
Chirayath et al. present a thorough investigation of the Auger electron spectrum of graphene on Cu utilizing time of flight spectroscopy to measure the energy of the emitted electrons. This is an interesting work, since it is the first experiment, where electrons emitted as a result of the filling of valence band holes (in contrast to core holes) could be identified in the spectrum. To overcome the problem of the large secondary background occurring when electron or photon beams are used to create valence band holes, the authors used positrons to create deep valence band holes via matter-antimatter annihilation. The experiments are complemented with first-principle calculations.

I suggest the publication, since the results are timely, the paper is well written and the analysis is conclusive. In fact, the manuscript leaves me with only two questions:

(1) The top panel of Fig.3(a) shows the ground state positron probability density which was determined by solving for the positron surface state wave function. Does this mean it is the wave function for a single positron, and if this is the case, would it change considerably if more positrons were present? Moreover, does the fact that the positrons are sent to the graphene sheet in a positron beam play a role? I assume, the situation can be imagined as follows: A single positron is sent to the graphene sheet, where it is trapped in a surface state (potential well in Fig.3(b)) until it annihilates an electron from the valence band of graphene, which happens before the next positron arrives. Is this picture correct?

(2) To calculate the Auger electron energy spectra, the energy conservation was taken into account. Was the momentum conservation considered as well, and if not, how would the results (the branching ratio in particular) change under the restrictions of momentum conservation?

Florian Wendler

Reviewer #3:
Remarks to the Author:
This highly creative work uses an exotic excitation source, positrons, to generate valence band holes in graphene and study their relaxation mechanism. Measurements of the emitted electron spectrum combined with modeling convincingly establish the hot-hole relaxation mechanism in graphene as an Auger transition involving the filling of these holes by other valence band electrons closer to the Fermi level and in most cases resulting in the emission of an Auger electron to the vacuum. The data, consistent with calculations, point to a probability of greater than 80% of the above Auger process occurring during hot-hole relaxation. These new insights into the electronic relaxation mechanisms of graphene are of wide fundamental and technological interest. Given that the claims of the paper are convincingly supported by the data presented (with some issues to be addressed below), I highly recommend its publication after the below comments are addressed.

1. The authors claim (page 7, last paragraph) that this hot-hole relaxation mechanism so prevalent in graphene should also be dominant in a broad class of solids (diamond, Si, Ge, Se, Te). However, this claim is unfounded. Hot-hole relaxation and Auger processes have been widely studied in Si and Ge, both in bulk and their nanocrystalline counterparts. It has been established, that quantum confinement effects play an important role in determining the rate and probability of these processes. For example, even though Auger recombination is the dominant relaxation mechanism in Ge and Si nanocrystals, intraband relaxation of single hot holes occurs primarily via phonon emission. I strongly suggest the authors remove this general claim.

2. On a minor note, W is most commonly used for work function, therefore, using it for the width of the valence band in the same equation that also contains the work function (denoted here by phi) can create confusion. I suggest denoting the work function with W (instead of phi) and using something else for the valence band width.
3. It would be very useful for the non-expert reader to define in one sentence what is meant by the VVV process.

Referee 1.

We thank Referee 1 for the general comments about the manuscript and for the useful suggestions and insights that will help to make the manuscript more clear.

1. “At page. 2 of the manuscript, version at my disposal, I do not entirely understand the term “elastic" used in the context of an Auger emission, which is actually an inelastic scattering by definition (“elastic emission of Auger electrons").”

   Answer:

   The term ‘elastic’ describes that, since Auger electron emission occurs from very near to the surface (a few monolayers), a fraction of the Auger electrons are ejected from the sample surface without any loss of energy. This helps in the identification of the chemical nature of the sample surface directly from the energy of the Auger peak. In order to avoid ambiguity we have changed the text in page 2, second paragraph, 3rd line to

   In the case of holes in core levels, the branching ratio for such a process is close to unity [1] and can result in the emission of electrons that can escape from the surface with no loss of energy.

2. “Still at page. 2, I would reconsider or, at least, soften the claim that “investigations of the spectra of Auger electrons emitted as a result of the filling of valence holes in solids have not been pursued".”

   Answer:

   Last sentence in page 2 has been reworded as.

   However, there have been only indirect investigations of the spectra of Auger electrons emitted as a result of the filling of valence-holes in solids [5, 6].

3. “Direct comparison between theoretical and “as recorded" experimental spectra without background subtraction has been already pursued in the past. Please cite the relevant

literature (e.g. see S Taioli et al. Phys. Rev. 493 (5), 237 (2010) and S. Taioli et al. Phys. Rev. B 79 (8), 085432 (2009)).”

Answer:

We have included the following text to second paragraph in page 2, line 8.

…and direct comparisons between theoretical line shapes and experimental spectra without background subtraction have been successful for core hole Auger spectra [3, 4].

4. “At page. 3 please consider to state more clearly what is meant by: “positron beam with an incident kinetic energy that is below the threshold for all impact induced electron emission". What exactly do the authors expect for higher energy positrons?”

   Answer:

   Above an incident kinetic energy of ~ 2 eV it is possible for positrons to excite secondary electron emission via a process in which the positron falls into a bound surface state (~ 2.5 eV deep) and gives all its energy to an electron near the Fermi level. We have added the threshold value (~ 2 eV) in the text and the reference, which discusses the above process, is placed next to the threshold value to make the sentence clearer.

   Recent measurements in our laboratory [9] have shown that it is possible to obtain Auger spectra that are completely free of such secondary electron background by utilizing a positron beam with an incident kinetic energy <u>below ~ 2 eV [10]</u>, the threshold for impact induced secondary electron emission.

5. “At page. 3 again please cancel out the parenthesis “(below 12 eV)". This number is indeed misleading, in view of the sentence reported immediately after, where the Auger peak occurs at 4 eV.”

   Answer:

   We have deleted the parenthesis.

6. (a) “At page. 6 please soften the claim that the agreement between experimental and calculated spectra is “excellent". In view of the level of theory used, which does not calculate explicitly the Auger matrix element, and superimposes many parametrized functions, such as the escape function. (b) Furthermore, the authors cannot claim that phonons do not contribute much to the broadening of the lineshape by simply comparing it with the experimental spectrum. Typically, these vibronic contributions do have an impact on the lineshape progression, possibly here are smeared out by the convolution with the superimposed broadening.”

(a) “At page. 6 please soften the claim that the agreement between experimental and calculated spectra is “excellent. In view of the level of theory used, which does not calculate explicitly the Auger matrix element, and superimposes many parameterized functions, such as the escape function.”

The sentence in page 6 is reworded as:

The comparison of the experimental and the theoretical VVV Auger line shapes (Fig. 4) shows that the calculated spectrum reproduces the overall width and shape of the experimental peak.

(b) “Furthermore, the authors cannot claim that phonons do not contribute much to the broadening of the lineshape by simply comparing it with the experimental spectrum.”

We have modified the text in page 7, indicating the possibility of phonons contributing to Auger line shape broadening.

While other mechanisms, e.g. phonons, may contribute to line shape broadening, our data indicate that the majority of the energy associated with the initial deep hole contributes to an Auger process.

7. “The method by Cini and Sawatzsky for calculating Auger lineshapes, using the convolution of the valence density of states, misses completely the information on the transition probability, which would require the calculation of the Auger matrix elements (and thus of the scattering wavefunction). So, this approach for calculating lineshapes, in addition to the authors' arbitrary choice of neglecting the hole-hole repulsion in the final states, is somehow closer to semi-empirical methods more than a fully ab-initio approach.”

Answer:

We agree with the referee that our calculation of the Auger line shape is not entirely ab-initio and we have so indicated in the revised text. First principle calculations were used in the determination of the electronic structure, positron wave function and the annihilation induced hole distribution. Once the distribution of the annihilation induced holes were determined ab-initio, we have used methods used by Lander et al. (*Phys. Rev.*, **91**, 1382 (1953)) and Hagstrum et al. (*Phys. Rev.*, **122**, 83 (1961)) who used a semi-empirical model of the electron escape probability to simulate the Auger line shape. To make the above structure of calculation clear to the reader we have made following changes to the text (changes are underlined) –

The 'first-principles calculations' in the first paragraph in the first page is changed to 'theoretical calculations'.

The first sentence of the last paragraph on page 5 is modified to (changes underlined)

Quantitative information regarding the VVV Auger process was obtained by comparing the experimentally obtained VVV Auger electron energy spectrum with the theoretical one. The calculation of the Auger line shape is based on a model similar to the one used for Auger neutralization of ions on solid surfaces [13, 14]. First, the electronic structure and the positron state for a free standing SLG and for SLG on Cu (111) were calculated from first-principles (details in the methods section and in SI)…

8. "At page. 6 and 7, I suppose the values in parenthesis (4 and 0.2) are meant to indicate errors. Please clarify better this point."
   Answer:
   Yes the value is the error on the ratio. To clarify this point better, we have included the symbol '±' in front of the error values.

9. "Please avoid the use of "hot holes" to describe sites that are active with respect to Auger transitions."
   Answer:
   We have removed the term "hot-holes" in the manuscript and substituted them with valence-holes or deep valence-holes.

10. "At page. 7, please clarify better the discussion about the assessment of the VVV Auger efficiency with respect to the KVV Auger lines. This is indeed almost an empirical way to deduce this."
    Answer:
    We have rewritten the discussion on page 7, to make clearer the method by which we estimate the VVV Auger efficiency from our data and theoretical calculations.

After correcting for the loss in the Auger peak intensities due to inelastic scattering (see SI), it was found that the measured ratio of the integrated intensity of the C VVV Auger peak to the integrated intensity of the C KVV Auger peak is 21±4. In order to estimate the branching ratio for the 'VVV' Auger decay of the deep valence-hole, we have compared this measured ratio to the theoretically calculated ratio. The integrated intensity of the C KVV Auger peak was theoretically calculated by assuming that all core holes created via positron-electron annihilation decay through an Auger process which is consistent with the previous results on the Auger

transition rates of K-shell holes in C [20]. The ratio of the calculated integrated intensities of the C VVV and C KVV Auger peaks is 20.7, assuming all of the valence band holes that are deep enough to result in electron emission into the vacuum undergo a VVV Auger transition. The calculated ratio is comparable to the experimental ratio if the branching ratio in the calculation was taken to be between 0.8 and 1 (where the range of the estimate was set by the uncertainty in the measurements of the integrated intensity ratio). Therefore, nearly all deep holes (between 80% -100%) in the valence band of graphene decay through an Auger process resulting in hole multiplication near the Fermi level. While other mechanisms, e.g. phonons, may contribute to line shape broadening, our data indicate that the majority of the energy associated with the initial deep hole contributes to an Auger process. Note that the higher intensity of the positron induced VVV Auger peak in comparison to the KVV Auger peak is primarily due to the fact that ~99% of the positrons annihilate with graphene valence electrons.

11. "In the discussion about the dissociative attachment at pag. 8, please cite the following reference (S Taioli et al. J. Phys. B: Atomic, Molecular and Optical Physics 39 (21), 4379 (2006)), which represents the first successful attempt of estimating the dissociation probability by using a wave packet approach in a complex triatomic potential."
    Answer:
    We have added the reference in the discussion about dissociative attachment of electrons resulting in DNA strand breaking.

12. "At page. 11 please correct the sentence stating "core holes in the valence band" as it is a bit misleading."
    Answer: The word "core" was a typo and we have deleted it from the sentence.

13. "In the Supporting Information, Electronic structure section, please add information about i) cell dimensions, possibly by adding a plot of the graphene-copper complex used in this work; ii) the energy cut-off."
    Answer:
    We have added the requested information and plot (Fig. S10) to the Supplementary Information

    The paragraph titled Electronic structure (page 13, Supplementary Information) is modified as (underlined portion is added)

    To obtain the electronic properties, we use the PAW method [6], as implemented in the VASP software package [7, 8, 9]. <u>The kinetic energy cutoff for the plane-wave expansion of the wave functions is set at 520 eV in all calculations. For the relaxation and ground state calculation of free</u>

standing graphene, a Γ-centered k-mesh of 15 x 15 is used, and is refined to 45 x 45 to calculate the DOS accurately. In the case of the ground state calculation and relaxation of graphene on top of the Cu (111) surface, a Γ - centered k-mesh of 21 x 21 is selected. In all calculations, the structures are relaxed until the forces on the atoms are smaller than 5 meV/Å. A vacuum of 15 Å is present in all slab models to avoid interaction between periodic images. Electron-electron exchange and correlation effects are described using the optB88-vdW functional [10] which includes long-range Van der Waals effects and yields accurate lattice constants for both Cu [11] and Graphite [12]. We find that this functional also reproduces a graphene - Cu interface spacing of 3.287 Å, which is in agreement with the value of 3.34 ± 0.06 Å obtained from total-reflection high-energy positron diffraction experiments [13]. Fig. 10 shows the unit cell used in the Cu (111) + graphene calculations.

14. “The numeration of some plots in the Supporting Information (S10, S11) looks not correct as well as the reference in the caption of the last picture of the Supporting Information.”
    Answer:
    We have corrected these errors.

Referee 2.

We thank Referee 2 for the comments about the manuscript, for recommending publication in Nature Communication and for the insightful questions which are addressed below.

1. “The top panel of Fig.3(a) shows the ground state positron probability density which was determined by solving for the positron surface state wave function. Does this mean it is the wave function for a single positron, and if this is the case, would it change considerably if more positrons were present? Moreover, does the fact that the positrons are sent to the graphene sheet in a positron beam play a role? I assume the situation can be imagined as follows: A single positron is sent to the graphene sheet, where it is trapped in a surface state (potential well in Fig. 3(b)) until it annihilates an electron from the valence band of graphene, which happens before the next positron arrives. Is this picture correct?”
   Answer:
   The picture presented by Referee 2 to describe the experiment is correct. The probability, for a second positron to arrive before the annihilation of the first one, is less than 1 ppm.

We have added the following sentence to the first paragraph on page 6 to clarify this point

“(with the beam intensity used in our experiment, the probability of a second positron arriving before the annihilation of the first one is very small, less than 1ppm and hence, there is only one positron on the surface at any time)”

If more than one positron is present on the surface, they will occupy the surface energy levels according to Fermi statistics. The overlap of the positron wave function with the surface electronic states will not change appreciably as positrons are delocalized parallel to the sample surface making the charge density due to the positrons on the surface negligible. An appreciable change from this simple picture would happen if the number of positrons existing simultaneously on the surface were on the order say of the concentrations of dopant impurities in a semiconductor. However, such high positron densities are presently not available in the laboratory.

2. “To calculate the Auger electron energy spectra, the energy conservation was taken into account. Was the momentum conservation considered as well, and if not, how would the results (the branching ratio in particular) change under the restrictions of momentum conservation?”

Answer:

As noted in the paper, we have used methods by Lander et al. (*Phys. Rev.*, **91**, 1382 (1953)) and Hagstrum et al. (*Phys. Rev*., **122**, 83 (1961)) to simulate the VVV Auger line shape. Our calculation has incorporated momentum conservation using the phenomenological approach of Hagstrum et al. (*Phys. Rev.*, **122**, 83 (1961)). This approach specifies the fraction of electrons that have a momentum component normal to the surface that is sufficient to overcome the surface barrier. A detailed calculation could change some details of the Auger line shape, but we do not expect such changes to have a significant effect on our estimate of the branching ratio that is based on the integrated intensity. More importantly a branching ratio of less than 0.8 does not account for the experimentally observed integrated intensity of the VVV Auger peak.

The empirical form of the escape probability is given in equation 20 of supplementary note 7. We have now added a sentence about the escape function in the main text.

Also, note that the momentum of the electron emitted via an Auger process is taken into account in the theoretical calculation of the line shapes and the integrated intensities of the Auger peaks using a phenomenological approach introduced by Hagstrum et al. [14].

Referee 3.

We thank Referee 3 for the comments about the manuscript, for recommending publication in Nature Communication and for the insightful suggestions which are addressed below.

1. "The authors claim (page 7, last paragraph) that this hot-hole relaxation mechanism so prevalent in graphene should also be dominant in a broad class of solids (diamond, Si, Ge, Se, Te). However, this claim is unfounded. Hot-hole relaxation and Auger processes have been widely studied in Si and Ge, both in bulk and their nanocrystalline counterparts. It has been established, that quantum confinement effects play an important role in determining the rate and probability of these processes. For example, even though Auger recombination is the dominant relaxation mechanism in Ge and Si nanocrystals, intraband relaxation of single hot holes occurs primarily via phonon emission. I strongly suggest the authors remove this general claim."

   Answer:

   We have changed the text to reflect the fact that VVV Auger transition is energetically possible in other wide valence band solids without commenting on the efficiency of the process.

   In page 4, the sentence -

   From equation (1) it can be seen that the maximum kinetic energy for such an electron emitted into the vacuum is $KE_{Auger-max}$ =W-ϕ, where W is the width of the valence band, and that electron can be emitted into the vacuum via a VVV transition for any material for which (W-ϕ)>0 (e.g. Si, Ge, Te, Se) [10, 11].

   has been modified to

   From equation (1) it can be seen that the maximum kinetic energy for such an electron emitted into the vacuum is:
   $KE_{Auger-max}$ =Δ_{VB}-ϕ , where Δ_{VB} is the Valence Band Width (i.e. the energy difference between the top and bottom of the filled states in the valence band). Equation (1) implies that it is energetically possible for an electron to be emitted into the vacuum via a VVV Auger transition from any material with Δ_{VB}> ϕ.

   On page 7, the first sentence in the last paragraph –

   Our work reveals that the emission of electrons as a result of Auger transitions involving the filling of holes in the valence band is highly efficient in a wide valence band solid (graphene) with branching ratio close to unity. Hence, the VVV Auger process will have

an important contribution to the low energy photoelectron or secondary electron spectra from graphene or from any solid with a wide valence band (diamond, Si, Se, Ge, Te etc.). The analysis of the intensity and shape of the low energy photoelectron and secondary electron spectra from wide band materials should consider the VVV Auger process provided the branching ratio of the VVV Auger process is significant.

has been modified to

Our work reveals that the emission of electrons as a result of Auger transitions involving the filling of deep holes in the valence band is highly efficient in graphene, with a branching ratio close to unity. Hence, VVV Auger processes can be expected to make an important contribution to the low energy photoelectron or secondary electron spectra from graphene.

2. "On a minor note, W is most commonly used for work function; therefore, using it for the width of the valence band in the same equation that also contains the work function (denoted here by phi) can create confusion. I suggest denoting the work function with W (instead of phi) and using something else for the valence band width."

   Answer:

   We have removed W from the equation and called it $\Delta_{VB,}$ the energy difference between the top and bottom of the filled states in the valence band

3. "It would be very useful for the non-expert reader to define in one sentence what is meant by the VVV process."

   Answer: We have modified the last sentence of the first paragraph in the 4th page to include a definition of VVV Auger transition.

   Following the nomenclature of Auger spectroscopy, we use the term VVV to refer to an Auger transition in which a hole in the valence band is filled by an electron higher up in the valence band, resulting in the ejection of a third electron from the valence band. Thus, a VVV Auger process starts with one hole in the valence band and ends with two valence band holes in the final state.

Reviewers' Comments:

Reviewer #1:
Remarks to the Author:
Dear Editor,
I believe that the authors accomplished satisfactorily the task of amending this article according to my suggestions. Thus, I warrant the publication of this work in Nature Communication.
As minor comments, please be aware that:

1) In the Supporting Information, in the paragraph titled "Electronic structure", the k-point grids used should be better reported as 15x15x1, 45x45x1, and 21x21x1 to avoid any possibile misunderstanding.
2) The correct surname in reference 20 is "Taioli S." and not "Taoli S." as erroneously reported.

Reviewer #2:
Remarks to the Author:
The paper is technically sound and the results are interesting. I can thus recommend the publication of this article.

Reviewer #3:
Remarks to the Author:
The authors have satisfactorily addressed all my comments. I recommend publication without further modifications.

Response to referees after second review

Referee 1.

We thank Referee 1 for recommending our manuscript for publication. We also thank the referee for the enlightening comments during the review.

1. In the Supporting Information, in the paragraph titled "Electronic structure", the k-point grids used should be better reported as 15x15x1, 45x45x1, and 21x21x1 to avoid any possibile misunderstanding.

   Answer: We thank the referee for pointing this out. We have made the necessary change in the supplementary information.

2. The correct surname in reference 20 is "Taioli S." and not "Taoli S." as erroneously reported.
   Answer: We thank the referee for pointing out the typo. We have corrected the surname.

Referee 2.

We thank Referee 2 for the comments about the manuscript, for recommending publication in Nature Communication and for the insightful questions during the review.

Referee 3.

We thank Referee 3 for the comments about the manuscript, for recommending publication in Nature Communication and for the insightful suggestions during the review.

Response to referees after first review

Dear Editor

We thank you for the kind examination of our manuscript titled- “Auger electron emission initiated by the creation of valence-band holes in graphene by positron annihilation”. We are glad to note that the referees find our work interesting with potential for publication in Nature Communication after the inclusion of corrections suggested and after answering some of the

concerns raised. We have addressed all the concerns raised by the referees and have included suggestions given by them as detailed below. The questions or suggestions are in color and in quotes while our reply is in black.

Referee 1.

We thank Referee 1 for the general comments about the manuscript and for the useful suggestions and insights that will help to make the manuscript more clear.

1. "At page. 2 of the manuscript, version at my disposal, I do not entirely understand the term "elastic" used in the context of an Auger emission, which is actually an inelastic scattering by definition ("elastic emission of Auger electrons")."
   Answer:
   The term 'elastic' describes that, since Auger electron emission occurs from very near to the surface (a few monolayers), a fraction of the Auger electrons are ejected from the sample surface without any loss of energy. This helps in the identification of the chemical nature of the sample surface directly from the energy of the Auger peak. In order to avoid ambiguity we have changed the text in page 2, second paragraph, 3rd line to

   In the case of holes in core levels, the branching ratio for such a process is close to unity [1] and can result in the emission of electrons that can escape from the surface with no loss of energy.

2. "Still at page. 2, I would reconsider or, at least, soften the claim that "investigations of the spectra of Auger electrons emitted as a result of the filling of valence holes in solids have not been pursued"."
   Answer:
   Last sentence in page 2 has been reworded as.

   However, there have been only indirect investigations of the spectra of Auger electrons emitted as a result of the filling of valence-holes in solids [5, 6].

3. "Direct comparison between theoretical and "as recorded" experimental spectra without background subtraction has been already pursued in the past. Please cite the relevant literature (e.g. see S Taioli et al. Phys. Rev. 493 (5), 237 (2010) and S. Taioli et al. Phys. Rev. B 79 (8), 085432 (2009))."
   Answer:
   We have included the following text to second paragraph in page 2, line 8.

...and direct comparisons between theoretical line shapes and experimental spectra without background subtraction have been successful for core hole Auger spectra [3, 4].

4. "At page. 3 please consider to state more clearly what is meant by: "positron beam with an incident kinetic energy that is below the threshold for all impact induced electron emission". What exactly do the authors expect for higher energy positrons?"
   Answer:
   Above an incident kinetic energy of ~ 2 eV it is possible for positrons to excite secondary electron emission via a process in which the positron falls into a bound surface state (~ 2.5 eV deep) and gives all its energy to an electron near the Fermi level. We have added the threshold value (~ 2 eV) in the text and the reference, which discusses the above process, is placed next to the threshold value to make the sentence clearer.

   Recent measurements in our laboratory [9] have shown that it is possible to obtain Auger spectra that are completely free of such secondary electron background by utilizing a positron beam with an incident kinetic energy <u>below ~ 2 eV [10]</u>, the threshold for impact induced secondary electron emission.

5. "At page. 3 again please cancel out the parenthesis "(below 12 eV)". This number is indeed misleading, in view of the sentence reported immediately after, where the Auger peak occurs at 4 eV."
   Answer:
   We have deleted the parenthesis.

6. (a) "At page. 6 please soften the claim that the agreement between experimental and calculated spectra is "excellent". In view of the level of theory used, which does not calculate explicitly the Auger matrix element, and superimposes many parametrized functions, such as the escape function. (b) Furthermore, the authors cannot claim that phonons do not contribute much to the broadening of the lineshape by simply comparing it with the experimental spectrum. Typically, these vibronic contributions do have an impact on the lineshape progression, possibly here are smeared out by the convolution with the superimposed broadening."

   > (a) "At page. 6 please soften the claim that the agreement between experimental and calculated spectra is "excellent. In view of the level of theory used, which does not calculate explicitly the Auger matrix element, and superimposes many parameterized functions, such as the escape function."

The sentence in page 6 is reworded as:

The comparison of the experimental and the theoretical VVV Auger line shapes (Fig. 4) shows that the calculated spectrum reproduces the overall width and shape of the experimental peak.

(b) "Furthermore, the authors cannot claim that phonons do not contribute much to the broadening of the lineshape by simply comparing it with the experimental spectrum."

We have modified the text in page 7, indicating the possibility of phonons contributing to Auger line shape broadening.

While other mechanisms, e.g. phonons, may contribute to line shape broadening, our data indicate that the majority of the energy associated with the initial deep hole contributes to an Auger process.

7. "The method by Cini and Sawatzsky for calculating Auger lineshapes, using the convolution of the valence density of states, misses completely the information on the transition probability, which would require the calculation of the Auger matrix elements (and thus of the scattering wavefunction). So, this approach for calculating lineshapes, in addition to the authors' arbitrary choice of neglecting the hole-hole repulsion in the final states, is somehow closer to semi-empirical methods more than a fully ab-initio approach."

Answer:

We agree with the referee that our calculation of the Auger line shape is not entirely ab-initio and we have so indicated in the revised text. First principle calculations were used in the determination of the electronic structure, positron wave function and the annihilation induced hole distribution. Once the distribution of the annihilation induced holes were determined ab-initio, we have used methods used by Lander et al. (*Phys. Rev.*, **91**, 1382 (1953)) and Hagstrum et al. (*Phys. Rev.*, **122**, 83 (1961)) who used a semi-empirical model of the electron escape probability to simulate the Auger line shape. To make the above structure of calculation clear to the reader we have made following changes to the text (changes are underlined) –

The 'first-principles calculations' in the first paragraph in the first page is changed to '<u>theoretical calculations</u>'.

The first sentence of the last paragraph on page 5 is modified to (changes underlined)

Quantitative information regarding the VVV Auger process was obtained by comparing the experimentally obtained VVV Auger electron energy spectrum with the theoretical one. The calculation of the Auger line shape is based on a model similar to the one used for Auger neutralization of ions on solid surfaces [13, 14]. First, the electronic structure and the positron state for a free standing SLG and for SLG on Cu (111) were calculated from first-principles (details in the methods section and in SI)…

8. "At page. 6 and 7, I suppose the values in parenthesis (4 and 0.2) are meant to indicate errors. Please clarify better this point."

   Answer:

   Yes the value is the error on the ratio. To clarify this point better, we have included the symbol '±' in front of the error values.

9. "Please avoid the use of "hot holes" to describe sites that are active with respect to Auger transitions."

   Answer:

   We have removed the term "hot-holes" in the manuscript and substituted them with valence-holes or deep valence-holes.

10. "At page. 7, please clarify better the discussion about the assessment of the VVV Auger efficiency with respect to the KVV Auger lines. This is indeed almost an empirical way to deduce this."

    Answer:

    We have rewritten the discussion on page 7, to make clearer the method by which we estimate the VVV Auger efficiency from our data and theoretical calculations.

After correcting for the loss in the Auger peak intensities due to inelastic scattering (see SI), it was found that the measured ratio of the integrated intensity of the C VVV Auger peak to the integrated intensity of the C KVV Auger peak is 21±4. In order to estimate the branching ratio for the 'VVV' Auger decay of the deep valence-hole, we have compared this measured ratio to the theoretically calculated ratio. The integrated intensity of the C KVV Auger peak was theoretically calculated by assuming that all core holes created via positron-electron annihilation decay through an Auger process which is consistent with the previous results on the Auger transition rates of K-shell holes in C [20]. The ratio of the calculated integrated intensities of the C VVV and C KVV Auger peaks is 20.7, assuming all of the valence band holes that are deep enough to result in electron emission into the vacuum undergo a VVV Auger transition. The calculated ratio is comparable to the experimental ratio if the branching ratio in the calculation was taken to be between 0.8 and 1 (where the range of the

estimate was set by the uncertainty in the measurements of the integrated intensity ratio). Therefore, nearly all deep holes (between 80% -100%) in the valence band of graphene decay through an Auger process resulting in hole multiplication near the Fermi level. While other mechanisms, e.g. phonons, may contribute to line shape broadening, our data indicate that the majority of the energy associated with the initial deep hole contributes to an Auger process. Note that the higher intensity of the positron induced VVV Auger peak in comparison to the KVV Auger peak is primarily due to the fact that ~99% of the positrons annihilate with graphene valence electrons.

11. "In the discussion about the dissociative attachment at pag. 8, please cite the following reference (S Taioli et al. J. Phys. B: Atomic, Molecular and Optical Physics 39 (21), 4379 (2006)), which represents the first successful attempt of estimating the dissociation probability by using a wave packet approach in a complex triatomic potential."
    Answer:
    We have added the reference in the discussion about dissociative attachment of electrons resulting in DNA strand breaking.

12. "At page. 11 please correct the sentence stating "core holes in the valence band" as it is a bit misleading."
    Answer: The word "core" was a typo and we have deleted it from the sentence.

13. "In the Supporting Information, Electronic structure section, please add information about i) cell dimensions, possibly by adding a plot of the graphene-copper complex used in this work; ii) the energy cut-off."
    Answer:
    We have added the requested information and plot (Fig. S10) to the Supplementary Information

    The paragraph titled Electronic structure (page 13, Supplementary Information) is modified as (underlined portion is added)

    To obtain the electronic properties, we use the PAW method [6], as implemented in the VASP software package [7, 8, 9]. <u>The kinetic energy cutoff for the plane-wave expansion of the wave functions is set at 520 eV in all calculations. For the relaxation and ground state calculation of free standing graphene, a Γ-centered k-mesh of 15 x 15 is used, and is refined to 45 x 45 to calculate the DOS accurately. In the case of the ground state calculation and relaxation of graphene on top of the Cu (111) surface, a Γ -centered k-mesh of 21 x 21 is selected.</u> In all calculations, the structures are relaxed until the forces on the atoms are smaller than 5 meV/Å. A vacuum of 15 Å is present in all slab models to avoid interaction between periodic

images. Electron-electron exchange and correlation effects are described using the optB88-vdW functional [10] which includes long-range Van der Waals effects and yields accurate lattice constants for both Cu [11] and Graphite [12]. We find that this functional also reproduces a graphene - Cu interface spacing of 3.287 Å, which is in agreement with the value of 3.34 ± 0.06 Å obtained from total-reflection high-energy positron diffraction experiments [13]. Fig. 10 shows the unit cell used in the Cu (111) + graphene calculations.

14. "The numeration of some plots in the Supporting Information (S10, S11) looks not correct as well as the reference in the caption of the last picture of the Supporting Information."

    Answer:

    We have corrected these errors.

Referee 2.

We thank Referee 2 for the comments about the manuscript, for recommending publication in Nature Communication and for the insightful questions which are addressed below.

1. "The top panel of Fig.3(a) shows the ground state positron probability density which was determined by solving for the positron surface state wave function. Does this mean it is the wave function for a single positron, and if this is the case, would it change considerably if more positrons were present? Moreover, does the fact that the positrons are sent to the graphene sheet in a positron beam play a role? I assume the situation can be imagined as follows: A single positron is sent to the graphene sheet, where it is trapped in a surface state (potential well in Fig. 3(b)) until it annihilates an electron from the valence band of graphene, which happens before the next positron arrives. Is this picture correct?"

    Answer:

    The picture presented by Referee 2 to describe the experiment is correct. The probability, for a second positron to arrive before the annihilation of the first one, is less than 1 ppm.

    We have added the following sentence to the first paragraph on page 6 to clarify this point

    "(with the beam intensity used in our experiment, the probability of a second positron arriving before the annihilation of the first one is very

small, less than 1ppm and hence, there is only one positron on the surface at any time)"

If more than one positron is present on the surface, they will occupy the surface energy levels according to Fermi statistics. The overlap of the positron wave function with the surface electronic states will not change appreciably as positrons are delocalized parallel to the sample surface making the charge density due to the positrons on the surface negligible. An appreciable change from this simple picture would happen if the number of positrons existing simultaneously on the surface were on the order say of the concentrations of dopant impurities in a semiconductor. However, such high positron densities are presently not available in the laboratory.

2. "To calculate the Auger electron energy spectra, the energy conservation was taken into account. Was the momentum conservation considered as well, and if not, how would the results (the branching ratio in particular) change under the restrictions of momentum conservation?"

   Answer:

   As noted in the paper, we have used methods by Lander et al. (*Phys. Rev.*, **91**, 1382 (1953)) and Hagstrum et al. (*Phys. Rev.*, **122**, 83 (1961)) to simulate the VVV Auger line shape. Our calculation has incorporated momentum conservation using the phenomenological approach of Hagstrum et al. (*Phys. Rev.*, **122**, 83 (1961)). This approach specifies the fraction of electrons that have a momentum component normal to the surface that is sufficient to overcome the surface barrier. A detailed calculation could change some details of the Auger line shape, but we do not expect such changes to have a significant effect on our estimate of the branching ratio that is based on the integrated intensity. More importantly a branching ratio of less than 0.8 does not account for the experimentally observed integrated intensity of the VVV Auger peak.

   The empirical form of the escape probability is given in equation 20 of supplementary note 7. We have now added a sentence about the escape function in the main text.

   Also, note that the momentum of the electron emitted via an Auger process is taken into account in the theoretical calculation of the line shapes and the integrated intensities of the Auger peaks using a phenomenological approach introduced by Hagstrum et al. [14].

Referee 3.

We thank Referee 3 for the comments about the manuscript, for recommending publication in Nature Communication and for the insightful suggestions which are addressed below.

1. "The authors claim (page 7, last paragraph) that this hot-hole relaxation mechanism so prevalent in graphene should also be dominant in a broad class of solids (diamond, Si, Ge, Se, Te). However, this claim is unfounded. Hot-hole relaxation and Auger processes have been widely studied in Si and Ge, both in bulk and their nanocrystalline counterparts. It has been established, that quantum confinement effects play an important role in determining the rate and probability of these processes. For example, even though Auger recombination is the dominant relaxation mechanism in Ge and Si nanocrystals, intraband relaxation of single hot holes occurs primarily via phonon emission. I strongly suggest the authors remove this general claim."

   Answer:

   We have changed the text to reflect the fact that VVV Auger transition is energetically possible in other wide valence band solids without commenting on the efficiency of the process.

   In page 4, the sentence -

   From equation (1) it can be seen that the maximum kinetic energy for such an electron emitted into the vacuum is $KE_{Auger\text{-}max} = W\text{-}\phi$, where W is the width of the valence band, and that electron can be emitted into the vacuum via a VVV transition for any material for which $(W\text{-}\phi)>0$ (e.g. Si, Ge, Te, Se) [10, 11].

   has been modified to

   From equation (1) it can be seen that the maximum kinetic energy for such an electron emitted into the vacuum is:
   $KE_{Auger\text{-}max} = \Delta_{VB}\text{-}\phi$, where Δ_{VB} is the Valence Band Width (i.e. the energy difference between the top and bottom of the filled states in the valence band). Equation (1) implies that it is energetically possible for an electron to be emitted into the vacuum via a VVV Auger transition from any material with $\Delta_{VB} > \phi$.

   On page 7, the first sentence in the last paragraph –

   Our work reveals that the emission of electrons as a result of Auger transitions involving the filling of holes in the valence band is highly efficient in a wide valence band solid (graphene) with branching ratio close to unity. Hence, the VVV Auger process will have an important contribution to the low energy photoelectron or secondary electron spectra from graphene or from any solid with a wide valence band (diamond, Si, Se, Ge, Te etc.). The analysis of the intensity and shape of the low energy photoelectron and secondary

electron spectra from wide band materials should consider the VVV Auger process provided the branching ratio of the VVV Auger process is significant.

has been modified to

Our work reveals that the emission of electrons as a result of Auger transitions involving the filling of deep holes in the valence band is highly efficient in graphene, with a branching ratio close to unity. Hence, VVV Auger processes can be expected to make an important contribution to the low energy photoelectron or secondary electron spectra from graphene.

2. "On a minor note, W is most commonly used for work function; therefore, using it for the width of the valence band in the same equation that also contains the work function (denoted here by phi) can create confusion. I suggest denoting the work function with W (instead of phi) and using something else for the valence band width."
   Answer:
   We have removed W from the equation and called it $\Delta_{VB,}$ the energy difference between the top and bottom of the filled states in the valence band

3. "It would be very useful for the non-expert reader to define in one sentence what is meant by the VVV process."
   Answer: We have modified the last sentence of the first paragraph in the 4th page to include a definition of VVV Auger transition.

   Following the nomenclature of Auger spectroscopy, we use the term VVV to refer to an Auger transition in which a hole in the valence band is filled by an electron higher up in the valence band, resulting in the ejection of a third electron from the valence band. Thus, a VVV Auger process starts with one hole in the valence band and ends with two valence band holes in the final state.
